# Self-Assessment of competence during post-graduate training in general medicine: A preliminary study to develop a portfolio for further education

**DOI:** 10.3205/zma001145

**Published:** 2017-11-15

**Authors:** Bert Huenges, Barbara Woestmann, Susanne Ruff-Dietrich, Herbert Rusche

**Affiliations:** 1 Ruhr-University Bochum, Department of General Medicine, Bochum, Germany; 2 Ruhr-University Bochum, JADE NRW, Bochum, Germany

**Keywords:** self-assessment, portfolio, specialist training, general medicine

## Abstract

Awareness of one’s own strengths and weaknesses is a key qualification for the specialist physician. We examined how physicians undergoing specialist training in general medicine rate themselves in different areas.

For this purpose, 139 participants receiving post-graduate training in general practice offered by the Medical Association of Westfalen-Lippe assessed themselves regarding their subjective confidence in 20 core competencies and 47 situations involving patient counseling in general practice. Their self-assessments were recorded on a five-point Likert scale. The study questions addressed acceptance and practicability of self-assessment, mean values, reliability, stratification and plausibility of the results in group comparison.

On average participants rated their subjective confidence with 3.4 out of 5 points. The results are self-consistent (Cronbach’s alpha >0.8), although there are considerable differences among competencies and among participants. The latter can be explained partly by biographical data, which supports the plausibility of the data.

Participants stated that regularly gathering data on subjective learning needs and the discussion of these needs with mentors and trainers contributes to improving their specialist training.

Elements for self-assessment are suitable for integration into a postgraduate training portfolio. These should be supplemented by formative assessment procedures.

## Zusammenfassung

Die Einschätzung der eigenen Kompetenz stellt eine Schlüsselqualifikation für den Facharzt^1^ dar. Wir haben untersucht, wie sicher sich Ärzte in Weiterbildung zum Facharzt für Allgemeinmedizin in unterschiedlichen Bereichen einstufen.

Dazu wurden 139 Selbsteinstufungen zur subjektiven Sicherheit in 20 Kernkompetenzen und 47 Beratungsanlässen von Teilnehmern (TN) allgemeinmedizinischer Weiterbildungskurse der Ärztekammer Westfalen-Lippe auf einer 5-stufigen Likert-Skala erfasst. Fragestellungen waren Akzeptanz und Praktikabilität, Mittelwert, Zuverlässigkeit, Streumaße und Plausibilität der Ergebnisse im Gruppenvergleich.

Ärzte in Weiterbildung stufen ihre subjektive Sicherheit im Mittel mit 3,4 von 5 Punkten ein. Die Ergebnisse sind in sich Konsistent. Dabei zeigen sich erhebliche Unterschiede – sowohl zwischen erfragten Kompetenzen als auch zwischen Teilnehmern. Letztere lassen sich zum Teil durch biographische Angaben der TN erklären, was die Plausibilität der Daten unterstützt.

Teilnehmer sind der Meinung, dass die regelmäßige Erhebung subjektiver Lernbedürfnisse und die Diskussion derselben mit Mentoren oder Weiterbildern einen Beitrag zur Verbesserung der Weiterbildung darstellt.

## 1. Introduction

Different competencies must be acquired in post-graduate education. Patterson et al. defined eleven areas of competence for general practitioners, including clinical expertise, professional integrity, empathy, communicative skills, conceptual thinking, and dealing with stressful situations [1].

We assume that each doctor pursuing specialized training has strengths and weaknesses regarding different competencies. Learners are only partially aware of their competencies, regardless of the extent to which they can be acquired or trained, and these competencies themselves can only be verified in part objectively.

Specialized medical training follows the rules of *deliberate practice* meaning that merely repeating activities is not sufficient to improve skills. Relevant are, among other things, feedback and the ability of the learner to recognize individual strengths and weaknesses in order to address them specifically [2]. In contrast to strictly regulated and organized undergraduate medical training, in Germany a great degree of responsibility rests on the learner to acquire the necessary competencies during post-graduate training.

Since maximum competency in all areas is desirable, but hardly realistic, doctors need to recognize their own limitations and assess their competencies in a reasonable way to reliably minimize the risk of errors and near misses at the expense of patients.

The competencies above can only be partially assessed in the final specialist examination (*Facharztprüfung*) using the common testing format in Germany. Alternative formats might improve assessment. Internationally, the trend is toward longitudinal, competence-based examination formats in post-graduate education in general practice and family medicine [3]. However, each format carries the risk that certain elements will not be captured leading to false positive or false negative results. This risk can be minimized by combining different assessment methods. Essential competencies that cannot be assessed using a given test format should be taken into account in a different way.

**Feedback** can help evaluate the self-perception of one's own competence [4]. Feedback is subjective and strongly dependent on the perception and experience of the observer. Feedback and assessments are required to promote realistic self-assessment of one's own competency.

In Germany, the five-year training to specialize in general practice encompasses certain rotations (in general, a minimum of two years of internal medicine in the hospital setting and another two years in the ambulatory setting) and a defined list of procedures to be performed before successful completion. These learning objectives have been increasingly criticized in recent years with voices calling for their supplementation with or replacement by competence-based approaches [5]. Furthermore, structured mentoring exists in only a few areas where mentors supervise further training and ensure that the competencies required for practice are acquired in different clinical rotations [6].

Several years ago, **logbooks** were introduced in post-graduate training which were meant to lead to regular conferences between trainers and trainees in order to verify acquired competencies and determine main areas of focus for upcoming training.

This study is based on the logbook for the post-graduate program in general practice at the University of Bochum (version 3.5), which is a supplement to the Medical Association’s official logbook containing items from several general medical curricula [7], [8], [9], [10], [11]. The main contents are 20 core competencies, specialist knowledge (27 items), practical skills (58 items), general medical consultations (47 items) and content from various medical specialist areas (122 items).

**Portfolios** go beyond the concept of logbooks. In addition to serving as elements to steer learning, they contain elements encouraging self-reflection and those for feedback and assessment [12].

In terms of personal quality control *(Have I learned everything I will need to know as a specialist physician?*) and as a basis for structured mentoring, a portfolio covering selected core competencies for future specialists in general medicine is to be developed. The model for this portfolio is based on regular self-assessment of the competencies required of specialists in general medicine [13].

This self-assessment serves the purpose of creating a (subjective) strength and weakness profile. The introduction of a reliable formative, longitudinal, work-based assessment can give learners the opportunity to reconsider their self-assessment and obtain a more realistic picture of their strengths and weaknesses [14].

In addition, self-assessments serve as a quality indicator at the collective level for the effectiveness of educational programs and advanced training [15].

**Research questions:**

1. Is the procedure feasible and is it accepted by doctors receiving post-licensure training?
2. What are the mean values, reliability and dispersion of the items?
3. Is the data plausible when compared to the group?

## 2. Methods

In total, 244 participants from six different courses in a training program for specialization in general practice were asked to rate their subjectively perceived confidence regarding 20 (general) medical core competences. The second survey was supplemented with a list of common consultation situations. In addition, sociodemographic variables (year of birth, year of medical licensing, years of medical practice as equivalent of full-time employment, advanced medical qualifications, experience in specialty areas, and current activity) were recorded.

The survey was carried out anonymously in paper-and-pencil format. The interviewees had the option to list an email address and receive individual feedback based on their own self-assessment as it compared to the mean values for all participants. Participants were also asked to provide qualitative feedback.

For further analysis, five different groups of participants were formed according to sociodemographic variables:

1. Specialization in general medicine took place immediately (time since graduation from medical school <7 years)
2. Second pathway (already completed training in another medical specialty)
3. Delayed specialization (time since graduation >7 years and medical activity >50% of the time)
4. Re-entry into the profession (same as III and medical activity <50% of the time)
5. Other/unknown.

Since there is a possibility to attend up to three training courses in any order, multiple participation in the survey was possible. When building the different groups, records that might have come from the same person were adjusted based on the personal data in order to avoid systematic distortions ("simple participants").

Analysis of the self-ratings was done using SPSS 24.

Using a multivariate regression, the maximum model which included all factors with a p value of <0,1 was used to test the independence of the variables and gradually reduce them to the minimum model. Here, only significant as well as relevant differences (OR<0,75 comparatively confident or OR>1,5 comparatively more confident) between the groups were evaluated as independent predictors. In the case of multiple participations, the first self-assessment ("single participant") was used as the basis.

A comparison of individual ratings from two interviews was possible in individual cases based on the email address given for feedback purposes. In order to determine the dynamics of self-assessment between the dates of the survey, the rating was compared in cases where it was clearly assigned to one person by means of the email address.

## 3. Results

In total, 139 participants (return rate 57%) completed the self-assessment of core competencies and 108 responses regarding the consultation situations.

For 44 data sets, multiple participations were assumed based on biographical data; 95 data sets showed such varied biographical information that it is clear they come from different people and were classified as “single participation” (see table 1 (Tab. 1)).

Participants pursuing the second pathway had a primary specialization in anesthesia (10), surgery or orthopedic surgery (8), psychiatry/neurology (3), pediatrics (2), occupational medicine, dermatology, radiology, radiotherapy and physical medicine/rehabilitation (1 each). 

"Others" are participants who cannot be clearly identified on the basis of their information (for example, general practitioners who take the course as a refresher or are pursuing another specialty).

The mean age for groups II and III differs only slightly, as do the years since graduation. However, with regard to professional experience (full-time equivalence of medical activity), the re-entering group (group IV), despite their higher average age, resembled the female doctors who are directly pursuing advanced training (group I).

Sixty-eight percent of participants had practical experience in internal medicine, followed by general medicine (56%), surgery (32%), anesthetics (15%), psychiatry (15%), neurology (7%), gynecology (6%), orthopedics (11%), geriatrics (5%), pediatrics (5%), dermatology (2%), or another area (13 fields with 1 entry each).

At the time of the survey, 55% of respondents were in practice, 15% in a hospital setting, 31% in other subject areas, two were already specialists in general medicine; those remaining are unknown.

On average, participants rated their subjective level of assurance in general medical core competencies on the scale from 1 (very unconfident) to 5 (very confident) as 3.6; in most cases (43% of responses), the "more confident" level (4 points) was chosen. The variance of the self-assessments is on average 0.8 (see table 2 (Tab. 2)). Cronbach’s alpha is 0.863 for these 20 items after exclusion of invalid cases.

General medical consultations were given on average 3.4 out of 5 points by the participants. The average variance of the self-ratings is 0.7 (see table 3 (Tab. 3)). Cronbach’s alpha is 0.938 for these 47 items after invalid cases are excluded.

Variables influencing self-assessment in multivariate analysis (single participation) are, in particular, previous professional experience in general medicine and surgery. Females are estimated to be more insecure on average, but the differences are only relevant for five counseling issues. Since the female participants in the sample also had less professional experience (full-time equivalent) than the male participants, the influence of the gender on the subjective of confidence in the items can only be assessed to a limited extent. Age and professional experience per se do not seem to have any significant independent influence (see table 4 (Tab. 4)).

Variance of individual self-assessments between two interviews could be compared in five cases:

On average after three to six months 35 of 67 items (min. 28, max. 43) were unchanged; participants felt more confident on 21 items (min. 10, max. 36) and less confident on 11 of 67 items (min. 3 max. 24) than at the time of the initial survey interview. Only five of the 67 items showed an unchanged assessment at the end of the survey period in all cases.

In the open-ended response section of the survey, many participants commented that they saw self-assessment as helpful in post-graduate education:

"*Yes, I feel it is helpful, not only in uncovering deficits, but also to assure oneself ... It is good to discuss existing deficits with my supervisor and hear how he sees my progress in comparison to my self-assessment...*.” (participant pursuing the direct path to a specialty in general practice).

*"I consider the list of the core competencies as good and sufficient to obtain an overall, but not too detailed overview. [...] The self-assessment at the end of the course regarding the counseling issues was once again helpful for me to identify weaknesses in more detail and concrete terms. Overall, I consider the self-assessment to be an important way to recognize weaknesses and actively improve them in the future. Personalized instruction is ideal, along with discussing relevant cases and standards in the current practice, which is the real task of every good instructor." *(Participant following the second pathway from anesthesia).

*"Consideration of the individual competencies was helpful for me in that the sheer variety of the core competences was made visible. It is possible to better structure post-graduate education since special attention can be placed on personal weak points in the daily routine (...)." *(Participant pursuing the second pathway from surgery).

## 4. Discussion

The method of self-assessment has proven to be practicable and is found to be helpful by individual participants. However, only 57% of those surveyed opted to use the self-assessment combined with individual feedback on their strengths and weaknesses relative to the comparative collective group.

The internal consistency of the self-assessment is comparatively high, so that a certain reliability of the test results in the collective as a whole can be assumed. The variability of the responses is comparatively high for many items – only five of 67 items showed no change in a sample size of five participants after three or six months.

### Influence of professional experience

Significant and relevant differences between groups of participants with different professional experiences and different survey dates suggest a certain plausibility of the results for the overall cohort. It is possible to identify trends and interesting differences between different subgroups which could not be determined otherwise without great effort using standard test procedures [15]. As expected, not only the amount of practical experience in general practice has a particularly large influence on the level of confidence regarding many items, but the amount of practical experience in surgery also seems to play a role for some items.

In the case of those aspiring to become general practitioners, it must be assumed that learning needs are extremely heterogeneous due to differing professional experience.

The influence of other possible independent factors, for instance gender, could not be determined due to the low number of participants. However, the multivariate analysis shows that the criteria "gender," "age" and "professional experience" compared with the individual’s academic background play a comparatively small role in self-assessment. Further studies of larger participant groups are needed on this aspect.

#### Needs assessment of doctors undergoing post-graduate training

Certain general trends can be seen. Most respondents feel confident performing physical examinations, mastering emergencies, establishing the doctor-patient relationship, taking care of documentation, engaging in self-study, and recognizing personal limits. Subjective uncertainties are most common concerning the care of chronically ill patients, palliative care, screening examinations, and the legal, organizational and business aspects of practicing general medicine.

While uncertainties are rarely indicated for some counseling issues (fever, vomiting, burning during urination, diarrhea, etc.), there are often uncertainties related to others (skin disorders, vaginal discharge, eye problems, etc.). Not to mention that psychogenic counseling, such as is the case for addiction, violence and sexual problems, was a cause of uncertainty in many participants.

Thus the analysis of the self-assessments can help to adapt the design and content of post-graduate training courses to match participant profile. In the case of training programs with meetings over a longer period of time, group-specific uncertainties can be tackled more specifically.

#### Individual needs assessment and dealing with strengths and weaknesses

A further focus of this survey is on the needs assessment of the individual learner. The goal of post-graduate education in general medicine cannot be to achieve maximum assurance on all issues. Rather, it is about becoming aware of one's own uncertainties in the diagnostic and therapeutic context and dealing with them in a targeted manner [16]. It should be noted that the individual’s view of his or her own competence and level of confidence may be different depending on personality (and gender).

Relative differences between individual items or the comparison group can be represented well in the format employed here: participants can become aware of the areas in which they see their subjectively greatest weaknesses and strengths and also see how they differ in comparison to other physicians undergoing training. This didactic element is intended to help doctors deal with specific topics in a targeted manner. Existing weaknesses should be identified to protect patients from a physician who overestimates his or her own knowledge or abilities. Here, participants can benefit from recognizing that they have over-estimated themselves in advance, either in terms of theoretical knowledge or practical skill. One goal of post-graduate education is to improve abilities to assess one's own strengths and weaknesses and to optimally steer further education.

#### Structuring continuing education

Self-assessment by participants is also conducive to individually tailoring the formative design of the training or monitoring the subjective gain in competence.

Such a survey provides an overview of what is fundamentally expected from a specialist in general medicine (see the comments above, also). While it is not possible to achieve a maximum level of assurance in all areas, important issues which are still the root of uncertainty in continuing education can be identified and actively addressed even within the scope of specialist testing (*Facharztprüfung*).

It must be borne in mind that over the course of professional practice, each physician has experiences which result in feelings of uncertainty, for instance, as he or she overlooks details while seemingly adhering to a routine of apparent safety. Accordingly, it can be that a greater level of uncertainty, as an expression of a more nuanced view of a particular topic, first occurs in the course of post-graduate education.

Self-assessment is complicated by the fact that the subjective level of confidence can level of confidence can decrease again as a result of the new context that comes with the transition from the stationary phase to the outpatient phase of post-graduate education. One reason for this could be that work in the low-prevalence area of general medicine, with its specific approach compared to the stationary setting, involves a relatively high degree of uncertainty [17], [18]. Further investigations are also required to explore this effect.

#### Strengths and weaknesses of the procedure

The self-assessment of one's own competence does not necessarily correspond to the objectively measured performance [19].

It is impossible to verify whether the assessments of the participants are reliable and accurate in individual cases. A comparison of self-estimations with the feedback given by trainers, peers or mentors, as well as results of (formative) work-based assessments are desirable. Approaches exist for pursuing further research.

By using an anonymized survey, the bias that comes with giving socially desirable answers has largely been avoided, but this effect must be taken into account when implementing a non-anonymous procedure.

The small sample of five participants, for which a follow-up observation was possible, gives an initial indication that the self-rating changes inter-individually over the course of post-graduate education; a much larger number of cases with individual gradients is needed for further analysis and evaluation of the effect sizes.

As a further limitation of the study, it should be noted that the self-assessment was carried out for pragmatic reasons on the basis of a regional logbook for post-graduate education in general medicine. Before the process can be further expanded with a larger number of cases, central tasks characterizing specialty training should be defined, for example, by formalized and concerted Entrusted Professional Activities (EPAs) [20].

Of central importance for general medicine is the extremely difficult to measure concept of hermeneutic case understanding that, among other factors which are equally difficult to measure such as "professionalism," is best captured, if at all, by means of multimodal approaches.

## 5. Summary and Outlook

The self-assessment of prescribed competencies is plausible for most of the participants and is experienced by individual participants as helpful.

The method of self-assessment is therefore basically suitable for inclusion in a portfolio for post-graduate education. A comparison of the self-assessment with the reference group can help participants question their own assessment in certain areas. The subjective self-rating should, however, be supplemented by structured feedback (from supervisors, mentors, patients, work-based assessments) if it is to be used as a formal aspect of continuing education programs.

Concentrating on core competencies could potentially help to avoid neglecting essential points and to provide support to participants wherever it seems necessary. The addition of concrete consultation situations allows for nuanced consideration of collective and individual strengths and weaknesses.

A national consensus-building would be desirable to identify which key core competencies are vital for the specialist and which other items (for example, in the form of EPAs) are worth striving for.

These ideas are based not only on the development of a portfolio for continuing education, but also the idea of repetitive self-assessment with a discussion of the results with a competent mentor. This could serve to adequately support "learning on the job" in continuing education [21] and to adapt the accompanying courses to the needs of the participants.

Education structured by such a portfolio could help to improve the educational situation in Germany.

## Competing interests

The authors declare that they have no competing interests. 

## Figures and Tables

**Table 1:**
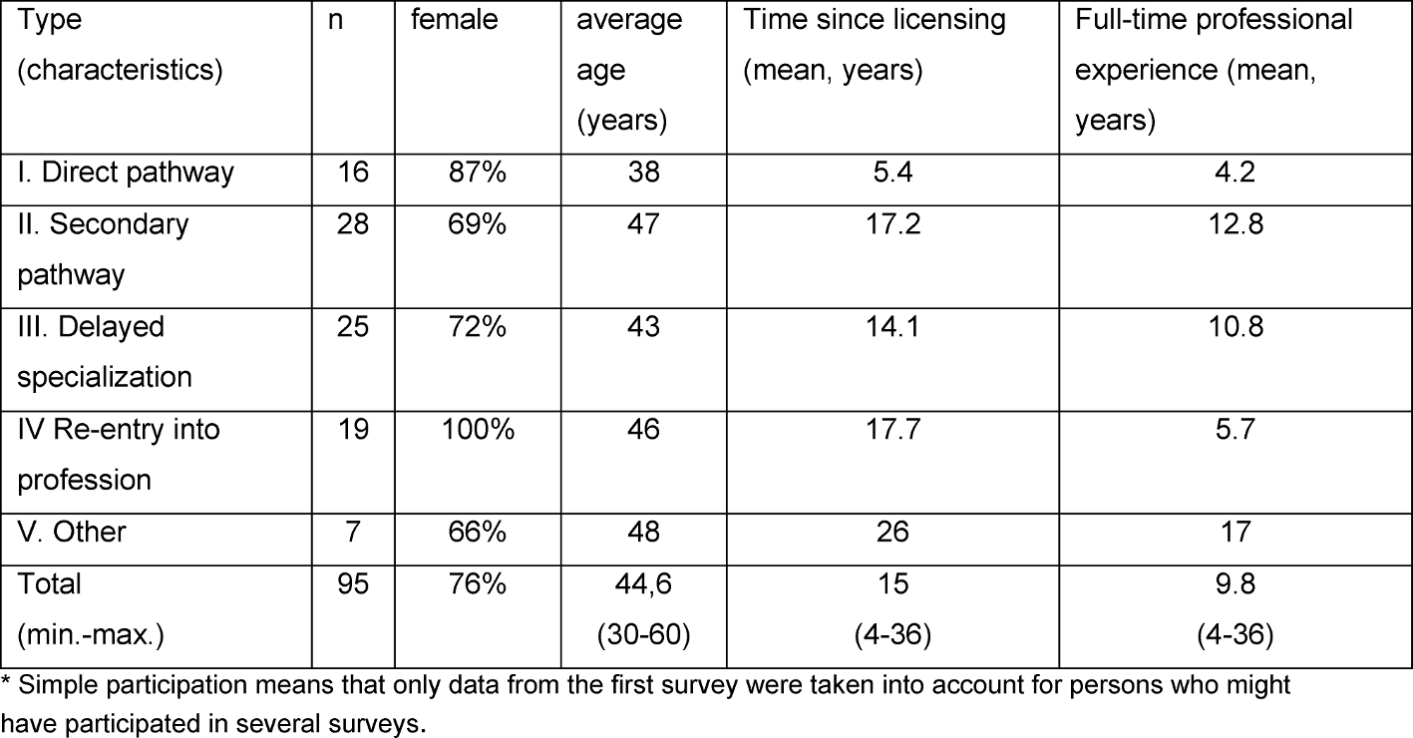
Characterization of the participants (single participation*)

**Table 2:**
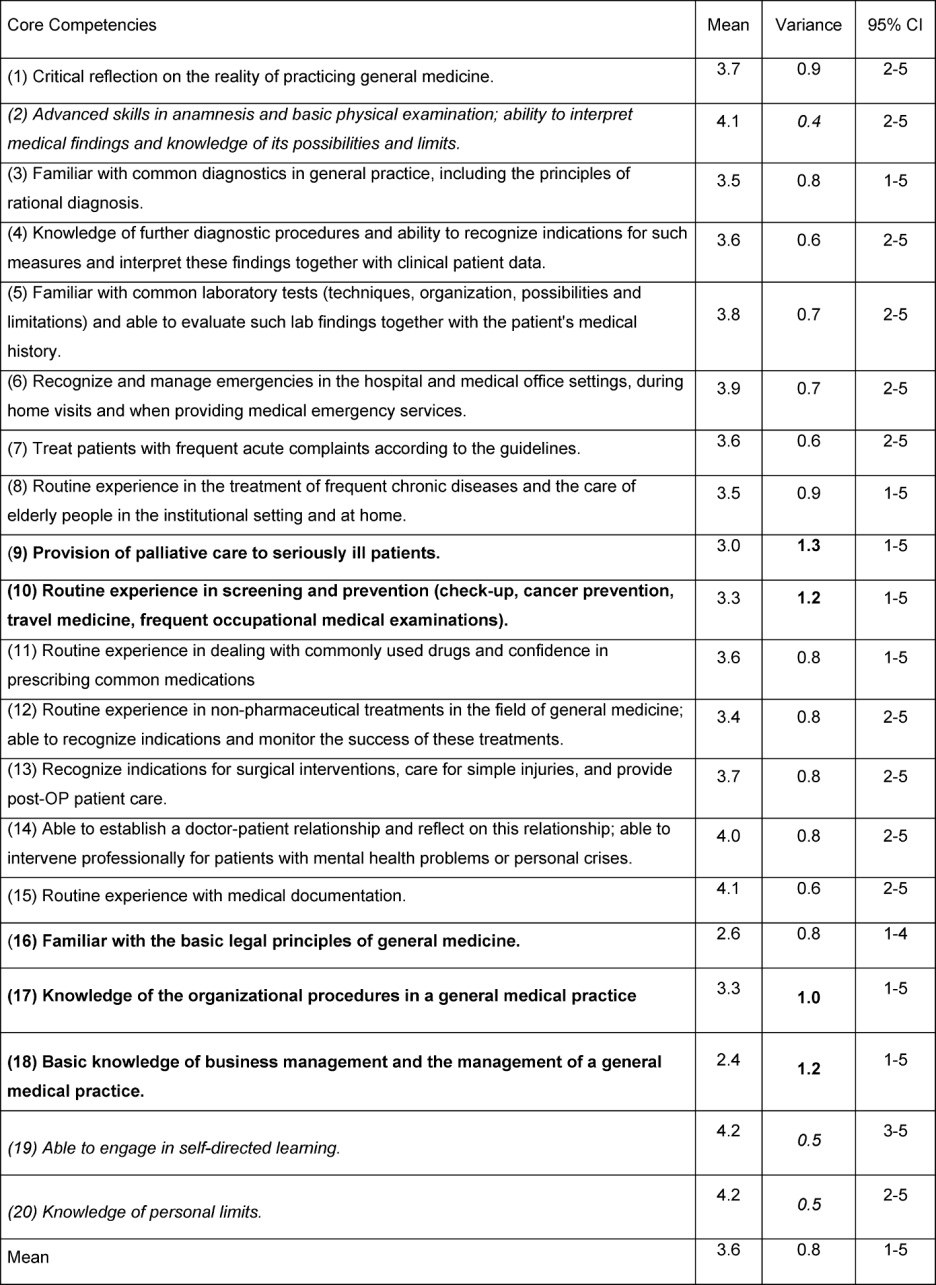
Self-assessment by 139 participants on general medical core competencies on a five-point Likert scale ranging from 1 (very uncertain) to 5 (very certain) Items for which the participants showed the most uncertainty (mean <3), and/or items with the greatest heterogeneity of assessment (variance ≥1) are in bold; Items with high mean for certainty (>4) and/or low variance (≤0.5) have been italicized.

**Table 3:**
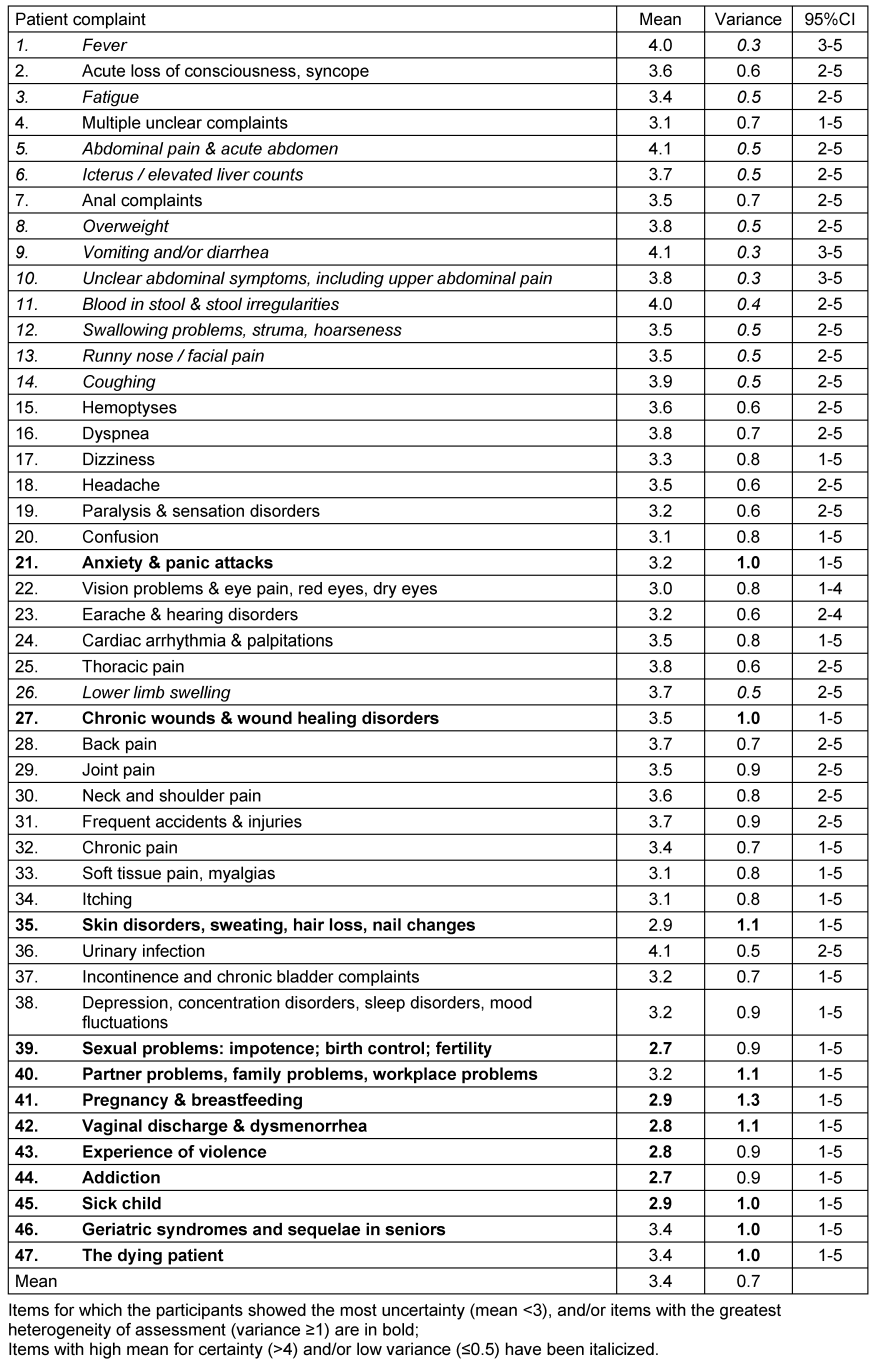
Self-assessment of 108 participants on consultations based on a five-point Likert scale ranging from 1 (very uncertain) to 5 (very certain)

**Table 4:**
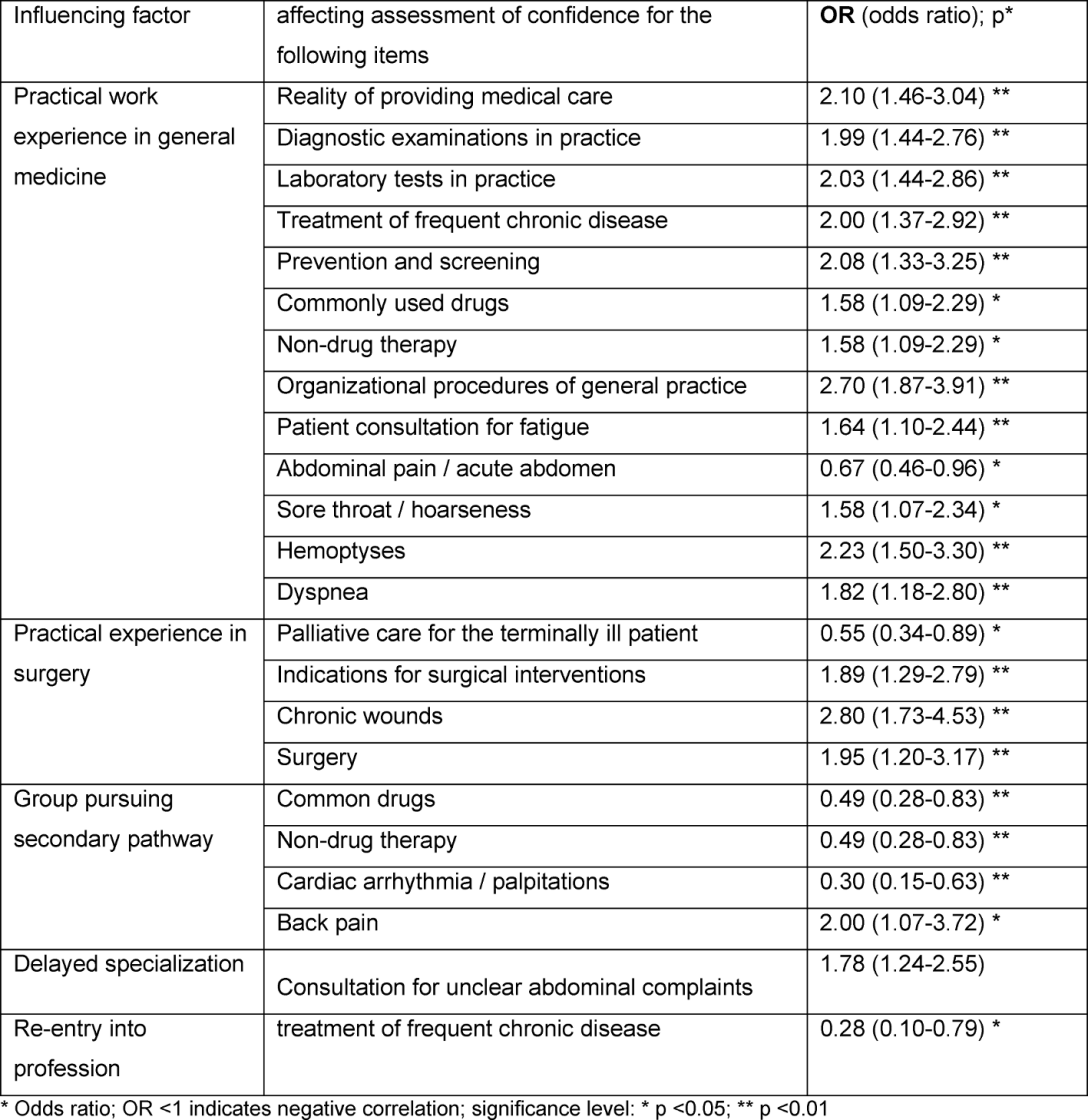
Significant and relevant biographical factors influencing self-assessment (Odds ratio; OR <1 indicates negative correlation) (n=95)

## References

[1] Patterson F, Ferguson E, Lane P, Farrell K, Martlew J, Wells A (2000). A competency model for general practice: implications for selection, training, and development. Br J Gen Pract.

[2] Duvivier RJ, van Dalen J, Muijtjens AM, Moulaert, Veronique RM, van der Vleuten CP, Scherpbier AJ (2011). The role of deliberate practice in the acquisition of clinical skills. BMC Med Educ.

[3] Flum E, Maagaard R, Godycki-Cwirko M, Scarborough N, Scherpbier N, Ledig T, Roos M, Steinhäuser J (2015). Assessing family medicine trainees--what can we learn from the European neighbours?. GMS Z Med Ausbild.

[4] Ende J (1983). Feedback in Clinical Medical Education. JAMA.

[5] David DM, Euteneier A, Fischer MR, Hahn EG, Johannink J, Kulike K, Lauch R, Lindhorst E, Noll-Hussong M, Pinilla S, Weih M, Wennekes V (2013). Die Zukunft der ärztlichen Weiterbildung in Deutschland - Positionspapier des Ausschusses Weiterbildung der Gesellschaft für Medizinische Ausbildung (GMA). GMS Z Med Ausbild.

[6] Broermann M (2014). Sinnvoll und vom Nachwuchs erwünscht: Mentoring in der Weiterbildung Allgemeinmedizin. Z Allgemeinmed.

[7] Ärztekammer Westfalen-Lippe (2009). Weiterbildungsordnung vom 09.04.2005, Allgemeinmedizin.

[8] Bernau R, Biesewig-Siebenmorgen J, Egidi G, Schmiemann G (2011). Ein 5-Jahres-Curriculum für die allgemeinmedizinische Fortbildung. Version 2010. Z Allgemeinmed.

[9] Bundesärztekammer (2015). (Muster-)Weiterbildungsordnung 2003.

[10] Bundesärztekammer (2015). (Muster-)Richtlinien über den Inhalt der Weiterbildung (MWBO 2003).

[11] Heyrman J (2005). European Academy of Teachers in General Practice (EURACT).

[12] Thomas D, Snadden M (2009). The use of portfolio learning in medical education. Med Teach.

[13] Sahu SK, Soudarssanane M, Roy G, Premrajan K, Sarkar S (2008). Use of Portfolio-based Learning and Assessment in Community-based Field Curriculum. Indian J Community Med.

[14] Cantillon P, Wood D (2010). ABC of learning and teaching in medicine.

[15] Davis DA, Mazmanian PE, Fordis M, van Harrison R, Thorpe KE, Perrier L (2006). Accuracy of physician self-assessment compared with observed measures of competence: a systematic review. JAMA.

[16] Biehn J (1982). Managing uncertainty in family practice. Can Med Assoc J.

[17] Donner-Banzhoff N (2012). Unsicherheit in der Allgemeinmedizin. Z Allgemeinmed.

[18] Wübken MH (2015). Umgang mit diagnostischer Unsicherheit in der hausärztlichen Praxis - eine Fragebogenkonstruktion.

[19] Eva KW, Regehr G (2005). Self-assessment in the health professions: a reformulation and research agenda. Acad Med.

[20] Ten Cate O (2006). Trust, competence, and the supervisor's role in postgraduate training. BMJ.

[21] Spencer J (2003). Learning and teaching in the clinical environment. BMJ.

